# Oxygen-18 isotope of breath CO_2_ linking to erythrocytes carbonic anhydrase activity: a biomarker for pre-diabetes and type 2 diabetes

**DOI:** 10.1038/srep08137

**Published:** 2015-01-30

**Authors:** Chiranjit Ghosh, Gourab D. Banik, Abhijit Maity, Suman Som, Arpita Chakraborty, Chitra Selvan, Shibendu Ghosh, Subhankar Chowdhury, Manik Pradhan

**Affiliations:** 1 Department of Chemical, Biological and Macromolecular Sciences, S. N. Bose National Centre for Basic Sciences, Salt Lake, JD Block, Sector III, Kolkata-700098, India; 2 Department of Endocrinology, Institute of Post Graduate Medical Education & Research and S.S.K.M Hospital, 244 A.J.C Bose Road, Kolkata-700020, India; 3 Department of Medicine, Vivekananda Institute of Medical Sciences, 99 Sarat Bose Road, Kolkata-700027, India; 4 Thematic Unit of Excellence on Nanodevice Technology, S. N. Bose National Centre for Basic Sciences, Salt Lake, JD Block, Sector III, Kolkata-700098, India

## Abstract

Carbonic anhydrase (CA), a well-characterized metalloenzyme, is associated with oxygen-18 ( ^18^O)-isotopic fractionations of CO_2_. To investigate how CA activity links the ^18^O of breath CO_2_ to pre-diabetes (PD) and type 2 diabetes (T2D) during metabolism, we studied pre- and post-dose CA activities in erythrocytes with simultaneous monitoring of ^18^O/ ^16^O-isotope ratios of breath CO_2_ and thereafter elucidated potential metabolic pathways underlying CA alteration in the pathogenesis of T2D. Here we show that the post-dose CA activity in both T2D and PD was markedly enhanced, whereas the non-diabetic controls (NDC) exhibited a considerable reduction in post-dose CA activity when compared with their basal CA activities. However, T2D and PD exhibited isotopic enrichments of ^18^O in breath CO_2_, while a marked depletion of ^18^O in CO_2_ was manifested in NDC. Thus, the isotopic enrichments and depletions of ^18^O in breath CO_2_ were well correlated with the changes in CA activities for controls, PD and T2D. Our findings suggest the changes in CA activities in erythrocytes may contribute to the pathogenesis of T2D and the breath C ^18^O ^16^O regulated by the CA activity as a potential biomarker for non-invasive assessment of T2D, and thus may open a new method for treating T2D.

Carbonic anhydrase (CA), a well-characterized pH-regulatory metalloenzyme found in most tissues including human erythrocytes (red blood cells), rapidly catalyzes the hydration of carbon dioxide (CO_2_) to form bicarbonate (HCO_3_^−^) and the reversible dehydration^1^,^2^. It also plays an important role in the transport of CO_2_ and ions (such as H^+^, Na^+^ and Cl^−^) along with pH-regulation in a variety of physiological processes ranging from respiration to intermediary metabolism at the cellular level^3^,^4^,^5^. Some early evidences suggest that the changes in CA activities in erythrocytes may be an initial step of altered metabolism in diabetes mellitus^2^,^6^. However, the precise role of CA activity, especially in the pathogenesis of type 2 diabetes mellitus (T2D), the most common deleterious metabolic disease at present worldwide^7^, is not currently known. Furthermore, the potential links between CA activity and T2D have not yet been fully elucidated.

Some early studies^8^,^9^,^10^ demonstrated that the oxygen-16 ( ^16^O) isotope in ^12^C ^16^O_2_ and the oxygen-18 ( ^18^O) isotope of body water (H_2_^18^O) are rapidly exchanged during the respiration process in humans, catalyzed by carbonic anhydrase. This efficient exchange suggests the possibility of exploiting the oxygen-isotope fractionations of CO_2_ in exhaled breath for non-invasive assessment of early-stage pre-diabetes prior to the onset of T2D. It also suggests a tantalizing hypothesis that monitoring stable ^18^O/^16^O isotope ratios of breath CO_2_ in response to CA activity may track the pathogenesis of the preclinical phase of T2D and hence may introduce a new strategy for treating T2D. Moreover, unravelling the exact metabolic pathways involved in causing the isotopic changes of ^12^C ^16^O ^18^O/^12^C ^16^O ^16^O in breath influenced by the enzymatic activity of CA in erythrocytes remains a challenge, whenever an individual is at high-risk for altered insulin action or for the acute onset of T2D.

In this study, we first investigated whether the total enzymatic activity of CA in erythrocytes is altered when individuals are in pre-diabetic and T2D states, and subsequently we assessed the precise role of CA activity in erythrocytes in response to glucose-stimulated insulin secretion that might influence the change in oxygen-isotope fractionations of CO_2_ in exhaled breath. We further explored the potential metabolic pathways underlying CA alteration in the pathogenesis of T2D and the mechanisms linking breath oxygen-isotopes to pre-diabetes (PD) and T2D.

## Results and Discussion

**Figure 1:**
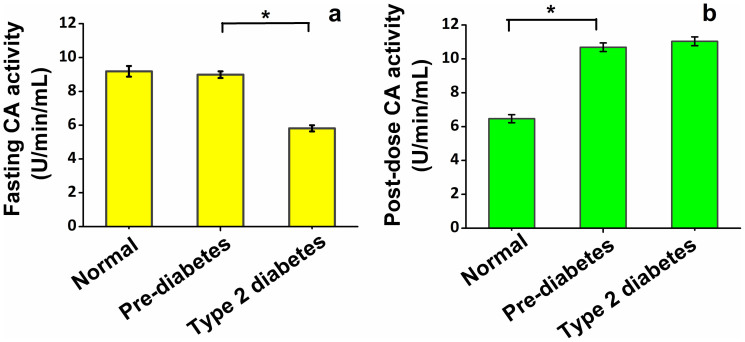
Erythrocyte CA activities associated with plasma glucose, insulin levels during the oral glucose tolerance test in non-diabetic control (NDC), pre-diabetes (PD) and type 2 diabetes (T2D). (a), Fasting CA activities [p < 0.01 for T2D (5.81 ± 0.18) versus PD (8.99 ± 0.19) and NDC (9.18 ± 0.31)]. (b), Post-dose CA activities [p = 0.34 for T2D (11.03 ± 0.26) as compared with PD (10.68 ± 0.25), whereas p < 0.01 among T2D, PD and NDC (6.47 ± 0.24)]. *p < 0.01. Data are means ± SEM.

To investigate the potential role of CA in the pathogenesis of T2D, we first tested whether the total basal CA activity altered in the pre-diabetic state and T2D after overnight fasting. We measured the total erythrocytes CA activity, which includes primarily three isozymes of CA (i.e. CAI, CAII and CAIII), spectro-photometrically in patients with non-diabetic controls (NDC) (n = 32), pre-diabetes (PD) (n = 39) and T2D (n = 48). In this investigation (Fig. 1a), individuals with T2D exhibited significantly lower basal CA activity as compared with PD and NDC, whereas no significant difference in basal CA activities was evident between subjects with PD and NDC. Several lines of evidence suggest that glycosylation of CA decreases its enzymatic and immunological activities and the glycosylation of CA is also enhanced during the peripheral circulation of erythrocytes^2^,^6^. Therefore, the decrease in basal CA activity for T2D individuals is possibly attributed to the increased level of glycosylation of the enzyme, caused by the exposure of erythrocytes to a higher concentration of plasma glucose compared to the PD and NDC (see Supplementary Fig. 1). Taken together, these findings indicate that the glycosylation plays a vital role in reduction of CA activity in individuals with T2D, whereas the glycosylation of CA does not play a significant role in either causing or facilitating the considerable differences in basal CA activities when an individual is in a normal or pre-diabetic state owing to their slight difference in blood glucose levels. Moreover, our results are coincidence with the previous study^11^, where the both CA I isozyme levels and total erythrocytes CA activities in fasting state have been reported to be downregulated in T2D as compared to control individuals.

We next explored the effect of CA activity in erythrocytes after administration of glucose, as the potential role of CA in response to glucose ingestion for NDC, PD and T2D remains unknown. To investigate this, we performed the 2h-oral glucose tolerance test (2h-OGTT). The post-dose CA activity (Fig. 1b), in both T2D and PD individuals, was markedly enhanced as compared with that of basal CA activity, although no significant difference of CA activities between these two groups was evident. In contrast, the NDC individuals exhibited a considerable reduction in post-dose CA activity when compared with basal CA activity. Erythrocyte receptor bound insulin has been proposed to alter the carbonic anhydrase activity^12^, suggesting that post-dose glucose stimulated plasma insulin levels may play an important role in changing the post-dose CA activities in erythrocytes in NDC, PD and T2D.

**Figure 2:**
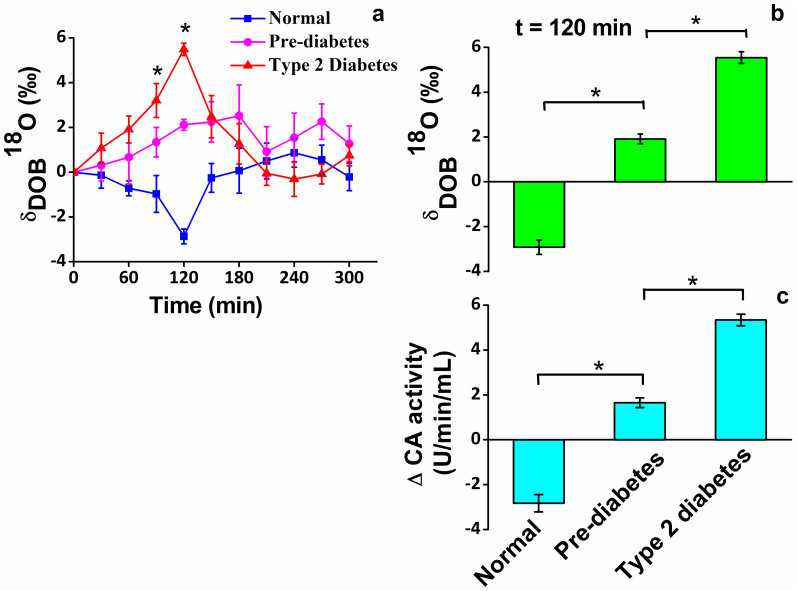
Erythrocyte ΔCA activity links with the δ_DOB_^18^O of breath CO_2_ for non-diabetic control (NDC), pre-diabetes (PD) and type 2 diabetes (T2D). (a), The maximum difference (p < 0.0001) of δ_DOB_^18^O (‰) was found at 120 min after glucose load. (b), (c), The ΔCA activities for T2D (5.34 ± 0.26), PD (1.65 ± 0.21) and NDC (−2.82 ± 0.38)] closely associated with δ_DOB_^18^O (5.54 ± 0.26, 1.91 ± 0.21 and −2.92 ± 0.32 for T2D, PD and NDC, respectively) in exhaled breath. *p < 0.01. Values are means ± SEM.

It is noteworthy that only pre-dose or post-dose erythrocyte CA activities do not allow one to distinguish the exact pathological state of the diabetes mellitus. We therefore, examined the absolute changes in CA activities (i.e. ΔCA) between the pre-dose (basal) and post-dose of glucose ingestion among all individuals (Fig. 2c). We found marked differences in ΔCA activities between subjects with NDC and PD, and subjects with PD and T2D, suggesting a potential link between changes in CA activities and altered metabolism responsible in individuals with PD and T2D. We have also established the previous hypothesis that the changes in CA activities in erythrocytes might signify the onset of altered metabolism in diabetes mellitus^2^. In view of this result, our findings suggest ΔCA activity in erythrocytes may contribute to the pathogenesis of T2D and might be considered as a potential biomarker for the detection of T2D.

**Figure 3:**
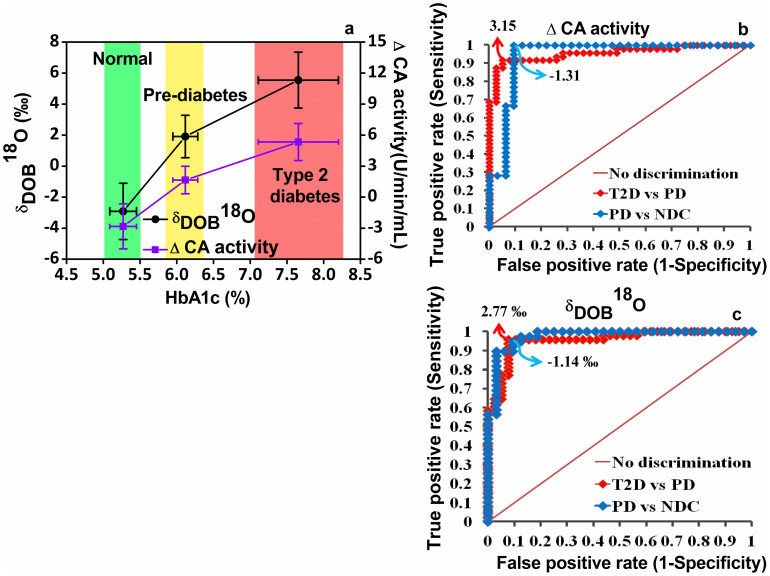
Distribution of ΔCA activities and δ_DOB_^18^O values against HbA1c (%) for non-diabetic control (NDC), pre-diabetes (PD) and type 2 diabetes (T2D). (a), Plot represents the clear transitions of ΔCA activities and δ_DOB_^18^O from NDC (green) to PD (yellow) and T2D (red). (b), (c), From receiver operating characteristic (ROC) curves analysis, the optimal diagnostic cut-off points were determined. The ΔCA activity <−1.31 for NDC; ΔCA activity >3.15 for T2D and −1.31 ≤ ΔCA activity ≤ 3.15 for PD, whereas δ_DOB_^18^O ‰ <−1.14 for NDC; δ_DOB_^18^O ‰ >2.77 for T2D and −1.14 ≤ δ_DOB_^18^O ‰ ≤ 2.77 for PD. Data are means ± SD.

We next investigated the oxygen-isotope fractionations of CO_2_ by monitoring the ^18^O/^16^O stable isotope ratios in exhaled breath, expressed as delta-over-baseline (DOB) relative to the Vienna Pee Dee Belemnite standard, i.e., δ_DOB_^18^O‰ = [(δ ^18^O‰)_t = t_ - (δ ^18^O‰)_t = basal_], associated with CA activities. To explore whether the changes in CA activities are associated with changes in ^18^O/^16^O isotope ratios of breath CO_2_, we investigated the excretion kinetics of δ_DOB_^18^O‰ values in breath samples from NDC, PD and T2D (Fig. 2a), using an optical cavity-enhanced integrated cavity output spectrometer. In this investigation, individuals with T2D exhibited significantly higher isotopic enrichments of ^18^O in CO_2_ compared with PD during the 2h-OGTT, while a marked depletion of ^18^O in CO_2_ was manifested in individuals with NDC (Fig. 2b). These outcomes suggest oxygen-isotope fractionations of CO_2_ in breath play an important role for non-invasive assessment of diabetes mellitus and hence these findings may open a new route to treat diabetes mellitus. However, the observations of isotopic depletion of ^18^O in CO_2_ for NDC and enrichments of ^18^O in CO_2_ for both individuals with T2D and PD are likely to be the effects of inhibition and promotion of catalytic activities of CA respectively, thus unveiling a potential link between CA activity in erythrocytes and the ^18^O-isotopic exchange in exhaled breath. As a result, we therefore posit that the monitoring of ^18^O-exchange between carbon dioxide (C ^16^O_2_) and body water (H_2_^18^O) influenced by the enzymatic activity of CA may distinctively track the evolution of pre-diabetes prior to the onset of T2D in a non-invasive way (Fig. 3a).

To investigate the precise transition from normal to pre-diabetes and then on to type 2 diabetes, we determined optimal diagnostic cut-off points of δ_DOB_^18^O‰ values in breath and ΔCA activity in erythrocytes using receiver operating characteristics curve (ROC) analysis (Fig. 3b and 3c). Individuals with δ_DOB_^18^O‰ > 2.77‰ and δ_DOB_^18^O‰ < −1.14‰ were considered T2D and NDC respectively, whereas subjects with 2.77 ≥ δ_DOB_^18^O‰ ≥ −1.14 were suggested to be PD and these corresponded to the diagnostic sensitivity and specificity of ~95% and ~91%, respectively. Conversely, a diagnostic cut-off point of ΔCA = 3.15 U/min/mL between individuals with PD and T2D, exhibited the sensitivity and specificity of 91.7% and 94.9%, respectively, whereas ΔCA = −1.31 U/min/mL correctly diagnosed individuals with NDC and PD corresponding to similar levels of diagnostic sensitivity and specificity. Taken together, these findings may have a broad clinical efficacy for accurate evaluation of diabetes mellitus either invasively or non-invasively and therefore provide a unique approach to treat the world's most common metabolic disease.

**Figure 4:**
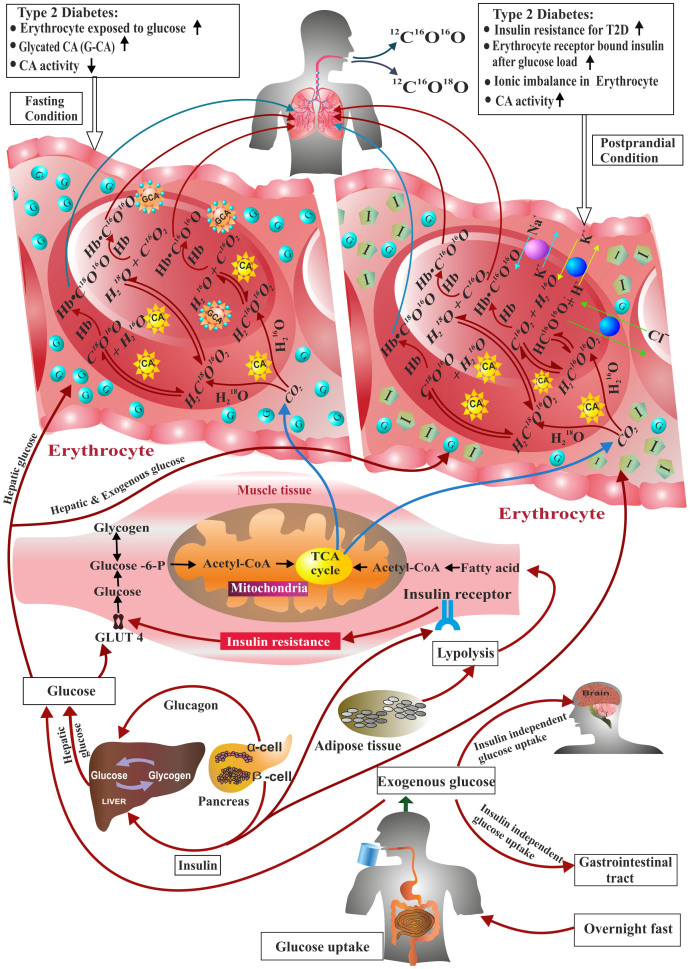
Potential metabolic pathways for ^18^O-isotopic exchange in response to CA activity. Under fasting condition, free fatty acids dispose predominantly in muscle tissues to form metabolite CO_2_, which enters into erythrocytes to form H_2_C ^18^O ^16^O_2_ (H_2_^18^O + CO_2_) and H_2_C ^16^O ^16^O_2_ (H_2_^16^O + CO_2_) compounds, catalyzed by CA activity. Erythrocytes of T2D are exposed to higher concentrations of glucose (G), which converts CA to GCA (glycosylated-CA), resulting in a substantial decrease of CA activity. Following the glucose load, the exogenous glucose stimulates insulin (I) production from the pancreatic islet. For PD and T2D, the insulin secretion increases in proportion to insulin resistance; elevated glycogenolysis, gluconeogensis and lipogenesis and diminished rate of insulin-dependent muscle tissue glucose uptake. When insulin action is suppressed for PD and T2D, there is a substantial increase in erythrocyte receptor-bound insulin, which enhances the CA activity. This alteration of CA activities, associated with insulin resistance during transition from NDC to T2D through PD, is reflected by the isotopic fractionation δ ^18^O in breath CO_2_.

Finally, we elucidated the potential metabolic pathways (Fig. 4) both in basal and postprandial (2h-OGTT) states underlying the mechanisms responsible for the alteration of CA activity and ^18^O-isotopic exchange in breath CO_2_. Under basal conditions, the majority of endogenous glucose disposal takes place in insulin-independent tissues like the brain, liver and gastrointestinal tract^13^,^14^. In T2D state, an elevated rate of hepatic glucose production is the major abnormality for the increased basal plasma glucose concentration^14^,^15^,^16^ and erythrocytes are then exposed to excessive glucose concentration which in turn makes a higher glycosylated form of CA than that in healthy individuals, resulting in reduction of total enzymatic activity of CA. However, in the basal state, more than 60% of cellular fuel for insulin-dependent muscle tissues is derived from free fatty acids for whole-body fuel oxidation^17^. The major metabolite CO_2_ produced by cellular oxidation reaches erythrocytes through the bloodstream and reacts with body water to form carbonic acid (H_2_CO_3_). The isotopes ^16^O of ^12^C ^16^O_2_ and ^18^O of H_2_^18^O are rapidly exchanged, catalyzed by CA activity, leading to the generation of H_2_C ^18^O ^16^O_2_ together with H_2_C ^16^O ^16^O_2._ All these carbonic acids rapidly degas to produce both C ^18^O ^16^O and C ^16^O ^16^O which subsequently bind to haemoglobin molecules to form carbaminohemoglobin compounds (Hb.CO_2_), which are then transported to the lungs where ^12^C ^16^O ^18^O and ^12^C ^16^O ^16^O are excreted in exhaled breath.

However, under postprandial condition, the exogenous glucose stimulates the release of insulin which facilities the majority of ingested glucose to uptake predominantly in peripheral tissues^18^ through the GLUT 4 transporter and a small fraction to adipose tissue. For T2D, the inhibition of insulin-receptor signalling due to insulin resistance and accordingly the lack of GLUT 4 recruitment plays a central role for the blunted rate of glucose transportation especially in insulin-dependent muscle tissues^15^. After glucose enters into muscle cell, it is phosphorylated to produce glucose-6-phosphate which later equilibrates between glycogenesis and glycolytic metabolic pathways. About one-third of the total glucose-6-phosphate, which enters into the glycolytic pathway, is predominantly oxidised into CO_2_ which is then transported to the erythrocyte and eventually excreted as ^12^C ^16^O ^18^O and ^12^C ^16^O ^16^O in breath. In case of pre-diabetes and newly diagnosed type 2 diabetes, the pancreas produces more insulin to compensate the insulin resistance. Therefore, erythrocyte is exposed to the environment of elevated receptor bound insulin in pre-diabetes and newly diagnosed type 2 diabetes, creating an ionic imbalance within the erythrocytes owing to increased proton production, Na^+^ ion uptake and possibly exchange of other ions such as K^+^ and Cl^−^ between plasma and erythrocytes^2^,^12^, leading to the enhancement of CA activity and accordingly alter the ^18^O-isotopic fractionations of CO_2_ in breath.

In conclusion, our findings point to a fundamental mechanism underlying the cause of altered CA activity in erythrocytes in individuals with dysglycemia. We have also taken a step towards unravelling the potential link between erythrocyte CA activity and ^18^O-isotopic fractionations of breath CO_2_, thus suggesting that ^18^O in CO_2_, the major metabolite of human breath, could be used as a potential molecular biomarker for the identification of accurate metabolic transition from normal to pre-diabetes and then on to type 2 diabetes in a non-invasive approach. Although many important gaps remain in our understanding of the exact metabolic pathways involved in causing the ^18^O-isotopic exchange and in the pathophysiology of T2D, our findings may open new perspectives in the molecular diagnosis of diabetes mellitus with broad clinical applications. Moreover, new insights into the mechanisms linking changes in CA activities to ^18^O-isotopic exchange are fostering exploration of the molecular basis of the pathogenesis of type 2 diabetes and new methods along with new pharmacological targets to prevent or treat the deleterious effects of this metabolic disease that threatens modern society.

## Methods

### Subjects

119 participants (n = 32 non-diabetic control, n = 39 pre-diabetes, n = 48 type 2 diabetes) were selected for the study. Individuals with hypertension, chronic respiratory disorder, interstitial lung disease, asthma or taking any type of medication that may alter the glucose metabolism are strictly excluded from the present study. Members from families with previous history of diabetes were also excluded from the study. Subjects were classified into three groups of type 2 diabetes (T2D), pre-diabetes (PD) and non-diabetic controls (NDC) according to the current criteria outlined by the American Diabetes Association^19^. Individuals with T2D were determined with glycosylated hemoglobin (HbA1c %) ≥ 6.5 and 2 hr glucose ≥ 200 mg/dL during an oral glucose tolerance test (OGTT), whereas PD was determined with 5.7 ≤ HbA1c (%) < 6.5 and 140 mg/dL ≤ 2 hr OGTT glucose < 200 mg/dL. Non-diabetic controls were defined with normal glucose tolerant, with 5.7 > HbA1c (%) and 2-hr OGTT glucose <140 mg/dL. The detailed subject characteristics are presented in Table 1. Informed consents were obtained from all participants according to the protocol approved by Institutional Ethics Committee of Post Graduate Medical Education & Research (IPGMER), Kolkata (Memo No. Inst/IEC/275) and the methods were carried out in accordance with the approved guidelines. All experimental protocols were also approved by the S.N. Bose Centre, Kolkata (Ref. No: SNB/PER-2-6001/13-14/1769).

### Study protocol

After overnight fasting (~12 hours), subjects were placed on chairs, and a baseline breath sample from each subject was taken in a breath sample collection bag (QUINTRON, USA, SL No.QT00892). A baseline blood sample (10 mL) was also collected simultaneously. A test meal containing 75-gm normal glucose dissolved in 150-mL water was orally administered and consumed within 1 minute. From this starting point, breath samples were collected in every 30-min interval, whereas blood samples were drawn after 2-hr of glucose load. During exhalation, subjects were instructed in such a way that they breathed normally, held for a few seconds and exhaled completely into a breath sample collection bag so that the oral breath first passed to dead space and the endogenously produced deep-breath (end expiratory breath) entered into the 750 mL reservoir bag through an one-way valve. The blood samples were utilized for measurement of glycated hemoglobin i.e. HbA1c (%), carbonic anhydrase activities and insulin levels. Breath samples were immediately analyzed to measure δ ^18^O of breath CO_2_.

### Biochemical analysis

Plasma Glucose concentrations were measured spectrophotometrically (2300 STAT Plus Glucose Analyzer). Insulin concentrations were measured by using monoclonal antibody coated immunoassay DIAsource INS-EASIA Kits (DIAsource ImmunoAssays S. A. Rue du Bosquet, 2, B-1348 Louvain-la-Neuve, Belgium). HbA1c (%) was determined in HPLC method by utilizing an HbA1c analyzer (D-10, USA).

### Breath sample analysis

Breath samples were drawn from the breath sample bags by an air-tight syringe (QUINTRON) through a T-connector fitted onto the reservoir bag. All breath samples were analyzed by a high-resolution isotopic CO_2_ integrated cavity output spectrometer (ICOS).

### Integrated Cavity Output Spectrometer

The cavity-enhanced absorption spectroscopy is an important tool for the measurement of trace gases along with isotopic species with ultra low concentrations. We utilized a high-resolution carbon dioxide isotope analyzer based on off-axis integrated cavity output spectroscopy (ICOS) exploiting a cavity-enhanced laser absorption technique to measure the isotopic compositions of CO_2_ of breath samples. There are few naturally occurring isotopes of carbon dioxide among which ^12^C ^16^O ^16^O and ^13^C ^16^O ^16^O are most abundant isotopes of CO_2_. The oxygen-18 isotope, i.e. ^12^C ^16^O ^18^O, is the stable third major naturally occurring isotope of CO_2_. The capability of the ICOS technique in comparison to conventional isotope ratio mass spectrometry (IRMS) to measure the stable isotope ratios of carbon dioxide ( ^12^C ^16^O ^16^O, ^13^C ^16^O ^16^O and ^12^C ^16^O ^18^O) has been well demonstrated elsewhere^20^,^21^. The ICOS instrument is so designed that laser light can be directed into the off-axis of the optical cavity. The present ICOS spectrometer (CCIA 36-EP, Los Gatos research, USA) consists of a continuous wave distributed feedback diode laser operating at ~2.05 μm and a high-finesse optical cavity (~59 cm long) with two high-reflectivity mirrors (R ~ 99.98%) at the ends of the measurement cell. This arrangement allows the laser light to move back and forth inside the cavity to reach an effective optical path-length of ~3 km and thus enabling high sensitivities. The laser frequency was repeatedly tuned to scan over 20 GHz across the P(36), R(28) and P(16) ro-vibrational lines to record the absorption spectra of ^12^C ^18^O ^16^O, ^12^C ^16^O ^16^O and ^13^C ^16^O ^16^O at the wave numbers of 4874.178 cm^−1^, 4874.448 cm^−1^ and 4874.086 cm^−1^ respectively, in the (2,0°,1) ← (0,0°,0) vibrational combination band of the CO_2_ molecule^22^. The transmitted laser intensities were recorded by exploiting a photodetector after passing through a breath sample of interest. Absorption was determined from the measurement of voltage from photodetector. Beer-Lambert law was utilized to calculate the concentration after integrating the absorption spectrum. The data were acquired at a rate of 1 Hz. The temperature of the cavity was maintained at 46°C by a resistive heater and feedback control system. The pressure of the cavity also regulated at 30 Torr by a diaphragm pump and solenoid valve in order to analyze the sample. The ^12^C ^18^O ^16^O enrichments in the breath samples have been expressed by the conventional notation, δ ^18^O in per mil (‰) relative to the standard Pee Dee Belemnite (PDB). It is described as below:

δ ^18^O‰ = (R_sample_/R_standard_ − 1) × 1000, where R_sample_ is the ^18^O/^16^O isotope ratio of the sample and R_standard_ is the international standard Vienna Pee Dee Belemnite value i.e. 0.0020672. The accuracy and precision for the δ ^18^O‰ measurements of the breath samples were determined by using a standard NOAA air tank. Accuracy was determined from the measurements of seven flasks filled from the certified standard NOAA air tank (Supplementary Table 1), whereas precision was determined from six consecutive measurements of same breath sample (Supplementary Table 2). We have determined that the typical precision of δ_DOB_^18^O ‰ [(δ ^18^O ‰)_2h__post-dose_ − (δ ^18^O ‰)_pre-dose_] measurements is ±0.18‰ in our current study.

### Blood sample preparation

10 mL of venous blood samples were collected from each participant in EDTA vacutainer tubes. The collected blood samples were centrifuged at 2000 r.p.m for 15 minutes. Plasma was isolated and buffy coat was removed. The red blood cells (RBC) were washed with 0.9% NaCl solution and allowed to spin against 4000 r.p.m for 20 minutes. The erythrocytes were lysed with ice-cold distilled water. The hemolysate was centrifuged at 10,000 r.p.m for 30 minutes to remove the ghost cells and the supernatant liquid was collected. The fresh supernatant liquid was used for analysis of carbonic anhydrase activity.

### Total erythrocyte carbonic anhydrase measurement

The esterase activity of carbonic anhydrase was determined spectro-photometrically as mentioned by Armstrong *et al.*^23^ with the modification described by Parui *et al.*^12^. In this assay, the esterase activity of carbonic anhydrase was determined spectro-photometrically from the hydrolysis rate of p-nitrophenyl acetate to p-nitrophenol. As the other esterases present in the hemolysate also hydrolyze the p-nitrophenyl acetate, a specific inhibitor of CA, acetazolamide was used and the acetazolamide-sensitive activity was taken as the net CA activity. This was performed from the differences between the absorbances before and after adding inhibitor. The protocol consisted of 100 μL hemolysate placed in 1 cm spectrometric cell containing 1.9 mL Tris buffer and 1 ml p-nitrophenyl acetate. The change of absorbance was measured over the period of 3 min at 348 nm before and after adding the hemolysate. The same measurements were performed in the presence of 0.1 mM acetazolamide inhibitor. The absorbance was measured by a UV-Vis spectrophotometer (Shimadzu UV-2600 Spectrophotometer). One unit of enzyme activity was expressed as μmol of p-nitrophenol relased/min/mL from hemolysate at room temperature (25°C)^2^,^12^.The following formula was used to calculate total erythrocyte CA activity:
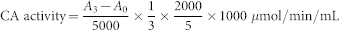
where A_3_ is absorbance after 3 min, A_0_ is the absorbance at 0 min, 5 × 10^3^ M^−1^cm^−1^ is the molar absorptivity of p-nitrophenol. The activity was normalized to 4.5 × 10^9^ cells/mL.

### Statistical method

Statistical analyses were performed using Origin Pro 8.0 (Origin Lab Corporation, USA) and Analyse-it Method Evaluation software (Analyse-it Software Ltd, UK, version 2.30). To compare normally distributed data, one way analysis of variance (ANOVA) was used whereas non-normal distributed data were analyzed by the Kruskal-Wallis test and Mann-Whitney test. A two sided p-value less than 0.05 was considered as statistically significant. All results were expressed as mean ± SEM except Fig. 3a, where values are mean ± SD. Receiver operating characteristic curves (ROC) were generated by plotting sensitivity (true positive rate) vs 1-specificity (false positive rate) to obtain the different cut-off points of δ_DOB_^18^O‰ values and ΔCA activities (Supplementary Table 4-Supplementary Table 7). The optimal cut-off value was considered to be that point which maximized the sensitivity and specificity (Supplementary Table 8). Data from insulin measurement were analyzed by MasterPlex software (Hitachi Software Engineering America, South San Francisco, CA, USA).

## Author Contributions

Funding and conception: M.P.; Study design: M.P. and C.G.; Overall supervision of the study: M.P., A.C., C.S., S.C. and S.G.; Collection and analysis of samples: C.G., G.D.B., A.M., S.S. and A.C.; Drafting of the manuscript with critical revision: All authors.

## Supplementary Material

Supplementary Information Supplementary Info

## Figures and Tables

**Table 1:** Clinical parameters of the study subjects. Data are expressed as mean ± SD. *Represents statistically significant differences (p < 0.05) among non-diabetic control (NDC), pre-diabetes (PD) and type 2 diabetes (T2D). The abbreviations M and F stand for male and female, respectively

Parameters	Non-diabetic control (NDC) (n = 32)	Pre-diabetes (PD) (n = 39)	Type 2 Diabetes (T2D) (n = 48)	p values
**Sex (M/F)**	13/19	14/25	19/29	
**Age (Years)**	35.94 ± 7.5	34.64 ± 6.5	38.6 ± 9.7	0.183
**Weight (kg)**	64.8 ± 6.5	65.9 ± 7	65.74 ± 5.7	0.748
**BMI (kg/m^2^)**	24.28 ± 2.8	24.17 ± 2.4	23.88 ± 2	0.877
**Fasting Plasma Glucose (mg/dL)**	90.19 ± 6.4	107.05 ± 5.1	140.42 ±7.7	<0.001*
**Fasting Plasma Insulin (μIU/mL)**	7.26 ± 3.4	9.6 ± 2.1	23.05 ± 3.2	<0.001*
**2-hr Post-dose Plasma Glucose (mg/dL)**	123.47 ± 10.1	172.56 ± 12.4	280.87 ± 39.1	<0.001*
**HbA1c (%)**	5.27 ± 0.2	6.11 ± 0.2	7.65 ± 0.5	<0.001*
**Fasting CA Activity (U/min/mL)**	9.18 ± 1.2	8.98 ± 1.2	5.81 ± 1.3	<0.001*
**Post-dose CA Activity (U/min/mL)**	6.47 ± 1.36	10.68 ± 1.6	11.03 ± 1.8	<0.001*
