# Supplementary material for: Oxygen-18 isotope of breath CO2 linking to erythrocytes carbonic anhydrase activity: a biomarker for pre-diabetes and type 2 diabetes
Source: Sci Rep. 2015 Jan 30;5:8137. doi: 10.1038/srep08137 (PMC4311236; doi:10.1038/srep08137)
Supplement: Supplementary Information — Supplementary Info [file srep08137-s1.pdf]

Supplementary information for

Oxygen-18 isotope of breath CO_2 linking to erythrocytes carbonic anhydrase activity: a biomarker for pre-diabetes and type 2 diabetes

Chiranjit Ghosh, Gourab D. Banik, Abhijit Maity, Suman Som, Arpita Chakraborty, Chitra Selvan, Shibendu Ghosh, Subhankar Chowdhury & Manik Pradhan*

*Corresponding Author. Email: manik.pradhan@bose.res.in

Supplementary Figure 1

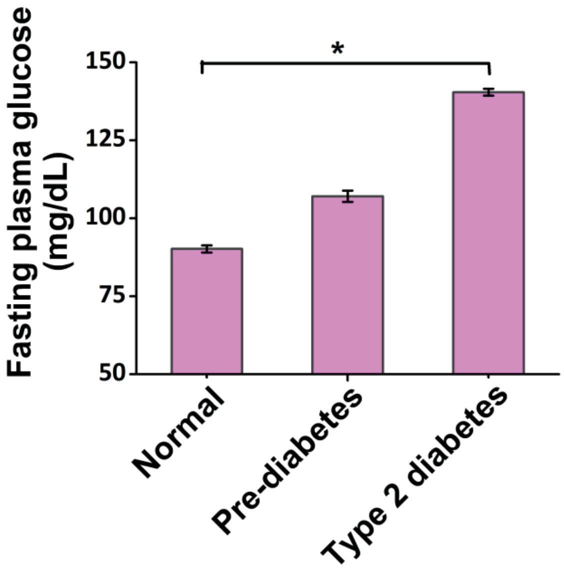

Figure 1: Fasting plasma glucose levels in non-diabetic control (NDC), pre-diabetes (PD) and type 2 diabetes (T2D): Fasting glucose [p<0.01for T2D (140.41 ± 1.12) versus PD (107.05 ± 1.81) and NDC (90.19 ± 1.13)]. *p<0.01. Data are means ± SEM.

Supplementary Table 1

Test Accuracy. Results of $\delta^{18}O$ (‰) measurements of seven flasks filled from a certified standard NOAA air tank (Serial No.CB10073).

**Flask**	**$\delta^{18}O$ (‰) measured by ICOS method**
Flask-1	-1.23
Flask-2	-1.09
Flask-3	-1.45
Flask-4	-0.67
Flask-5	-1.18
Flask-6	-0.91
Flask-7	-1.13
Avg.	-1.09
NOAA	-1.00
**Diff.**	0.09

Supplementary Table 2

Test precision of $\delta_{DOB}{}^{18}O$ ‰. To calculate the precision, two breath samples (pre-dose and post-dose) were taken from a diabetic participant and analyzed by the ICOS spectrometer. After six consecutive measurements of each set (pre-dose and post-dose samples), the precision was calculated from the standard error of $\delta_{DOB}{}^{18}O$ values from six sets of measurements.

$(\delta^{18}O)_{measurement}$	Difference ($\delta_{DOB}{}^{18}O$ ‰) measured by ICOS method
$(\delta^{18}O)_{test1}$-$(\delta^{18}O)_{test2}$ ‰	4.81
$(\delta^{18}O)_{test3}$-$(\delta^{18}O)_{test4}$ ‰	4.32
$(\delta^{18}O)_{test5}$-$(\delta^{18}O)_{test6}$ ‰	5.33
$(\delta^{18}O)_{test7}$-$(\delta^{18}O)_{test8}$ ‰	4.58
$(\delta^{18}O)_{test9}$-$(\delta^{18}O)_{test10}$ ‰	5.25
$(\delta^{18}O)_{tes11}$-$(\delta^{18}O)_{test12}$ ‰	5.39
	Precision= ± 0.18

Supplementary Table 3

Summary of data for 119 individuals. The datasheet includes $\delta_{DOB}{}^{18}O$ (‰) and ΔCA activity (U/min/mL) values of non-diabetic control (NDC), pre-diabetes (PD) and type 2 diabetes (T2D).

Subjects	Sex	Age (years)	HbA1c (%)	Metabolic Disorder	ΔCA activity (U/min/mL)	$\delta_{DOB}{}^{18}O$ (‰)
N1	M	31	5.0	NDC	-4.02	-4.94
N2	F	46	5.0	NDC	-1.55	-2.04
N3	F	44	5.0	NDC	-4.69	-4.65
N4	F	31	5.0	NDC	-2.97	-4.61
N5	F	33	5.1	NDC	-3.23	-1.88
N6	M	28	5.1	NDC	-1.52	-4.66
N7	M	43	5.1	NDC	-3.18	-2.61
N8	F	31	5.1	NDC	-2.41	-1.74

N9	M	33	5.6	NDC	2.55	-2.22
N10	M	26	5.2	NDC	-1.35	-2.82
N11	F	27	5.2	NDC	-1.54	1.75
N12	F	24	5.6	NDC	1.32	-0.44
N13	F	29	5.3	NDC	2.57	0.59
N14	F	33	5.3	NDC	-2.26	-1.58
N16	M	30	5.1	NDC	-3.39	-3.31
N17	M	38	5.3	NDC	-1.41	-2.45
N18	F	37	5.5	NDC	-1.95	-1.48
N19	M	39	5.4	NDC	-3.57	-2.48
N20	M	43	5.3	NDC	-3.98	-3.48
N21	F	44	5.5	NDC	-1.32	-2.47
N22	M	48	5.5	NDC	-5.06	-1.14
N23	M	41	5.5	NDC	-2.77	-3.15
N24	F	47	5.3	NDC	-2.03	-2.66
N25	M	35	5.2	NDC	-5.08	-4.72
N26	M	25	5.2	NDC	-5.22	-2.77
N27	F	29	5.4	NDC	-2.61	-3.45
N28	M	42	5.4	NDC	-5.42	-4.84
N29	M	35	5.5	NDC	-6.86	-5.42
N30	M	33	5.1	NDC	-5.28	-6.41
N31	M	39	5.2	NDC	-5.25	-5.79
N32	M	30	5.3	NDC	-3.38	-3.93
P1	M	41	5.9	PD	1.68	-1.55
P2	M	26	6.4	PD	2.39	-0.65
P3	M	31	6.0	PD	2.44	5.27
P4	F	38	6.0	PD	1.4	3.96
P5	M	37	6.0	PD	1.61	-0.86
P6	F	43	6.1	PD	0.91	1.18
P7	M	45	6.1	PD	3.11	-1.19
P8	F	44	6.2	PD	0.91	2.56
P9	M	30	6.2	PD	3.05	2.56

P10	F	25	6.3	PD	3.15	1.11
P11	M	28	6.3	PD	1.81	1.67
P12	F	34	6.4	PD	3.04	1.73
P13	F	39	5.8	PD	1.78	2.48
P14	M	43	5.9	PD	2.59	2.68
P15	M	37	5.9	PD	0.17	4.88
P16	F	32	6.1	PD	1.30	2.74
P17	M	45	6	PD	1.71	2.63
P18	F	32	6.3	PD	1.52	2.67
P19	F	29	6.2	PD	1.64	0.88
P20	F	26	6.1	PD	1.54	1.72
P21	M	27	6.4	PD	2.9	1.95
P22	M	36	6.3	PD	3.26	1.99
P23	M	34	5.9	PD	4.39	2.01
P24	F	42	5.8	PD	3.11	2.33
P25	M	37	5.9	PD	-1.18	1.45
P26	M	29	6	PD	-1.01	1.74
P27	F	27	6.1	PD	2.16	1.86
P28	M	26	6.2	PD	2.25	2.77
P29	M	33	6.3	PD	2.65	1.56
P30	M	37	6	PD	2.39	1.08
P31	M	28	6.1	PD	0.77	2.44
P32	F	24	6.3	PD	-1.26	2.04
P33	M	30	6.2	PD	-0.5	1.46
P34	M	38	6.1	PD	0.29	2.45
P35	M	34	5.9	PD	0.72	1.57
P36	M	35	6.2	PD	3.03	1.42
P37	F	47	6.3	PD	2	2.76
P38	M	44	6.1	PD	-0.28	2.6
P39	M	38	6.2	PD	1.04	2.58
D1	F	47	6.7	T2D	4.22	2.09
D2	M	45	6.8	T2D	1.04	7.8

D3	M	35	6.9	T2D	4.43	4.42
D4	M	29	6.8	T2D	3.23	6.59
D5	F	46	7.3	T2D	3.23	5.62
D6	M	43	7.8	T2D	4.16	5.18
D7	M	30	8.2	T2D	4.42	6.88
D8	M	28	8.1	T2D	4.76	6.19
D9	M	44	7.6	T2D	5.35	4.73
D10	M	34	7.6	T2D	7.37	3.7
D11	M	49	8.2	T2D	1.73	6.81
D12	F	53	8.1	T2D	7.54	6.48
D13	F	54	7.7	T2D	5.26	5.23
D14	M	41	7.6	T2D	5.9	7.87
D15	M	27	7.3	T2D	4.35	6.21
D16	F	34	7.1	T2D	3.38	7.05
D17	F	52	7.2	T2D	5.19	8.87
D18	M	54	7.6	T2D	4.89	3.45
D19	M	24	8.4	T2D	6.22	6.11
D20	F	38	8.2	T2D	6.9	5.43
D21	M	35	8.6	T2D	7.34	6.49
D22	F	31	7.1	T2D	6.71	5.84
D23	F	42	7.3	T2D	3.94	3.54
D24	M	53	7.6	T2D	7.07	6.01
D25	M	50	7.4	T2D	6.12	1.83
D26	F	47	7.2	T2D	5.27	2.88
D27	M	34	7.5	T2D	4.38	3.2
D28	F	26	8.1	T2D	2.59	4.14
D29	M	31	6.9	T2D	4.12	3.23
D30	F	27	6.6	T2D	6.24	4.12
D31	M	24	6.8	T2D	3.63	7.56
D32	F	34	8.5	T2D	5.56	2.81
D33	M	49	8	T2D	4.34	7.2
D34	M	53	8.1	T2D	8.15	2.9

D35	F	33	8.4	T2D	9.34	3.55
D36	M	51	8.2	T2D	5.61	4.23
D37	M	50	8.3	T2D	6.53	5.03
D38	M	47	8.1	T2D	4.52	6.25
D39	F	25	8.3	T2D	5.37	7.02
D40	M	26	8.2	T2D	7.09	6.74
D41	M	34	7.9	T2D	8.69	8.64
D42	F	33	7.7	T2D	4.65	4.54
D43	M	48	7.5	T2D	2.44	8.11
D44	F	37	7.3	T2D	5.7	6.55
D45	F	33	7	T2D	7.06	7.42
D46	M	27	7.6	T2D	6.97	6.11
D47	F	29	7.9	T2D	7.93	5.48
D48	M	37	8.1	T2D	5.26	8.01

Supplementary Table 4

Data analysis from Receiver operating characteristic curve (ROC) to obtain the optimal diagnostic cut-off value of ΔCA activity (U/min/mL) between Type 2 diabetes (T2D) and pre-diabetes (PD). The selected optimal cut-off values for CA activity (U/min/mL) have been highlighted. TP : True positive rate, FP : False positive rate, TN : True negative rate, FN : False negative rate.

T2D vs PD	TP	FP	TN	FN	Sensitivity	Specificity	Predictive Value (+)	Predictive Value (-)
-1.25	48	38	1	0	1.000	0.026	0.56	1.00
-1.18	48	37	2	0	1.000	0.051	0.56	1.00
-1.005	48	36	3	0	1.000	0.077	0.57	1.00
-0.50	48	35	4	0	1.000	0.103	0.58	1.00
-0.27	48	34	5	0	1.000	0.128	0.59	1.00
0.16	48	33	6	0	1.000	0.154	0.59	1.00
0.29	48	32	7	0	1.000	0.179	0.60	1.00

0.72	48	31	8	0	1.000	0.205	0.61	1.00
0.76	48	30	9	0	1.000	0.231	0.62	1.00
0.90	48	29	10	0	1.000	0.256	0.62	1.00
0.91	48	28	11	0	1.000	0.282	0.63	1.00
1.03	47	28	11	1	0.979	0.282	0.63	0.92
1.04	47	27	12	1	0.979	0.308	0.64	0.92
1.30	47	26	13	1	0.979	0.333	0.64	0.93
1.40	47	25	14	1	0.979	0.359	0.65	0.93
1.51	47	24	15	1	0.979	0.385	0.66	0.94
1.54	47	23	16	1	0.979	0.410	0.67	0.94
1.60	47	22	17	1	0.979	0.436	0.68	0.94
1.64	47	21	18	1	0.979	0.462	0.69	0.95
1.68	47	20	19	1	0.979	0.487	0.70	0.95
1.71	47	19	20	1	0.979	0.513	0.71	0.95
1.72	46	19	20	2	0.958	0.513	0.71	0.91
1.78	46	18	21	2	0.958	0.538	0.72	0.91
1.80	46	17	22	2	0.958	0.564	0.73	0.92
2.003	46	16	23	2	0.958	0.590	0.74	0.92
2.15	46	15	24	2	0.958	0.615	0.75	0.92
2.25	46	14	25	2	0.958	0.641	0.77	0.93
2.38	46	13	26	2	0.958	0.667	0.78	0.93
2.39	46	12	27	2	0.958	0.692	0.79	0.93
2.43	46	11	28	2	0.958	0.718	0.81	0.93
2.44	45	11	28	3	0.938	0.718	0.80	0.90
2.58	45	10	29	3	0.938	0.744	0.82	0.91
2.59	44	10	29	4	0.917	0.744	0.81	0.88
2.65	44	9	30	4	0.917	0.769	0.83	0.88
2.89	44	8	31	4	0.917	0.795	0.85	0.89
3.03	44	7	32	4	0.917	0.821	0.86	0.89
3.03	44	6	33	4	0.917	0.846	0.88	0.89
3.05	44	5	34	4	0.917	0.872	0.90	0.89
3.10	44	4	35	4	0.917	0.897	0.92	0.90

3.11	44	3	36	4	0.917	0.923	0.94	0.90
3.15	44	2	37	4	0.917	0.949	0.96	0.90
3.22	43	2	37	5	0.896	0.949	0.96	0.88
3.23	42	2	37	6	0.875	0.949	0.95	0.86
3.26	42	1	38	6	0.875	0.974	0.98	0.86
3.37	41	1	38	7	0.854	0.974	0.98	0.84
3.63	40	1	38	8	0.833	0.974	0.98	0.83
3.93	39	1	38	9	0.813	0.974	0.98	0.81
4.12	38	1	38	10	0.792	0.974	0.97	0.79
4.15	37	1	38	11	0.771	0.974	0.97	0.78
4.22	36	1	38	12	0.750	0.974	0.97	0.76
4.34	35	1	38	13	0.729	0.974	0.97	0.75
4.35	34	1	38	14	0.708	0.974	0.97	0.73
4.37	33	1	38	15	0.688	0.974	0.97	0.72
4.39	33	0	39	15	0.688	1.000	1.00	0.72
4.41	32	0	39	16	0.667	1.000	1.00	0.71
4.42	31	0	39	17	0.646	1.000	1.00	0.70
4.52	30	0	39	18	0.625	1.000	1.00	0.68
4.65	29	0	39	19	0.604	1.000	1.00	0.67
4.75	28	0	39	20	0.583	1.000	1.00	0.66
4.89	27	0	39	21	0.563	1.000	1.00	0.65
5.18	26	0	39	22	0.542	1.000	1.00	0.64
5.25	25	0	39	23	0.521	1.000	1.00	0.63
5.25	24	0	39	24	0.500	1.000	1.00	0.62
5.26	23	0	39	25	0.479	1.000	1.00	0.61
5.35	22	0	39	26	0.458	1.000	1.00	0.60
5.37	21	0	39	27	0.438	1.000	1.00	0.59
5.56	20	0	39	28	0.417	1.000	1.00	0.58
5.60	19	0	39	29	0.396	1.000	1.00	0.57
5.70	18	0	39	30	0.375	1.000	1.00	0.57
5.90	17	0	39	31	0.354	1.000	1.00	0.56
6.12	16	0	39	32	0.333	1.000	1.00	0.55

6.22	15	0	39	33	0.313	1.000	1.00	0.54
6.23	14	0	39	34	0.292	1.000	1.00	0.53
6.53	13	0	39	35	0.271	1.000	1.00	0.53
6.71	12	0	39	36	0.250	1.000	1.00	0.52
6.89	11	0	39	37	0.229	1.000	1.00	0.51
6.96	10	0	39	38	0.208	1.000	1.00	0.51
7.05	9	0	39	39	0.188	1.000	1.00	0.50
7.07	8	0	39	40	0.167	1.000	1.00	0.49
7.09	7	0	39	41	0.146	1.000	1.00	0.49
7.34	6	0	39	42	0.125	1.000	1.00	0.48
7.36	5	0	39	43	0.104	1.000	1.00	0.48
7.53	4	0	39	44	0.083	1.000	1.00	0.47
7.93	3	0	39	45	0.063	1.000	1.00	0.46
8.14	2	0	39	46	0.042	1.000	1.00	0.46
8.69	1	0	39	47	0.021	1.000	1.00	0.45
9.33	0	0	39	48	0.000	1.000	-	0.45

Supplementary Table 5

Data analysis from Receiver operating characteristic curve (ROC) to obtain the optimal diagnostic cut-off value of ΔCA activity (U/min/mL) between pre-diabetes (PD) and non-diabetic control (NDC). The selected optimal cut-off value has been highlighted. TP: True positive rate, FP : False positive rate, TN : True negative rate, FN : False negative rate.

PD vs NDC	TP	FP	TN	FN	Sensitivity	Specificity	Predictive Value (+)	Predictive Value (-)
-6.86	39	31	1	0	1.000	0.031	0.56	1.00
-5.41	39	30	2	0	1.000	0.063	0.57	1.00
-5.27	39	29	3	0	1.000	0.094	0.58	1.00
-5.25	39	28	4	0	1.000	0.125	0.58	1.00
-5.21	39	27	5	0	1.000	0.156	0.59	1.00
-5.07	39	26	6	0	1.000	0.188	0.60	1.00

-5.05	39	25	7	0	1.000	0.219	0.61	1.00
-4.69	39	24	8	0	1.000	0.250	0.62	1.00
-4.01	39	23	9	0	1.000	0.281	0.63	1.00
-3.97	39	22	10	0	1.000	0.313	0.64	1.00
-3.57	39	21	11	0	1.000	0.344	0.65	1.00
-3.50	39	20	12	0	1.000	0.375	0.66	1.00
-3.39	39	19	13	0	1.000	0.406	0.67	1.00
-3.38	39	18	14	0	1.000	0.438	0.69	1.00
-3.23	39	17	15	0	1.000	0.469	0.70	1.00
-3.17	39	16	16	0	1.000	0.500	0.71	1.00
-2.96	39	15	17	0	1.000	0.531	0.72	1.00
-2.77	39	14	18	0	1.000	0.563	0.74	1.00
-2.61	39	13	19	0	1.000	0.594	0.75	1.00
-2.40	39	12	20	0	1.000	0.625	0.77	1.00
-2.26	39	11	21	0	1.000	0.656	0.78	1.00
-2.03	39	10	22	0	1.000	0.688	0.80	1.00
-1.95	39	9	23	0	1.000	0.719	0.81	1.00
-1.55	39	8	24	0	1.000	0.750	0.83	1.00
-1.54	39	7	25	0	1.000	0.781	0.85	1.00
-1.51	39	6	26	0	1.000	0.813	0.87	1.00
-1.40	39	5	27	0	1.000	0.844	0.89	1.00
-1.35	39	4	28	0	1.000	0.875	0.91	1.00
-1.31	39	3	29	0	1.000	0.906	0.93	1.00
-1.25	38	3	29	1	0.974	0.906	0.93	0.97
-1.18	37	3	29	2	0.949	0.906	0.93	0.93
-1.005	36	3	29	3	0.923	0.906	0.92	0.91
-0.50	35	3	29	4	0.897	0.906	0.92	0.88
-0.27	34	3	29	5	0.872	0.906	0.92	0.85
0.16	33	3	29	6	0.846	0.906	0.92	0.83
0.29	32	3	29	7	0.821	0.906	0.92	0.80
0.72	31	3	29	8	0.795	0.906	0.91	0.78
0.76	30	3	29	9	0.769	0.906	0.91	0.76

0.90	29	3	29	10	0.744	0.906	0.91	0.74
0.91	28	3	29	11	0.718	0.906	0.90	0.72
1.04	27	3	29	12	0.692	0.906	0.90	0.71
1.30	26	3	29	13	0.667	0.906	0.90	0.69
1.31	26	2	30	13	0.667	0.938	0.93	0.70
1.40	25	2	30	14	0.641	0.938	0.93	0.68
1.51	24	2	30	15	0.615	0.938	0.92	0.66
1.54	23	2	30	16	0.590	0.938	0.92	0.65
1.60	22	2	30	17	0.564	0.938	0.92	0.64
1.64	21	2	30	18	0.538	0.938	0.91	0.62
1.68	20	2	30	19	0.513	0.938	0.91	0.61
1.71	19	2	30	20	0.487	0.938	0.91	0.60
1.78	18	2	30	21	0.462	0.938	0.90	0.59
1.80	17	2	30	22	0.436	0.938	0.90	0.57
2.003	16	2	30	23	0.410	0.938	0.89	0.56
2.15	15	2	30	24	0.385	0.938	0.88	0.55
2.25	14	2	30	25	0.359	0.938	0.88	0.54
2.38	13	2	30	26	0.333	0.938	0.87	0.53
2.39	12	2	30	27	0.308	0.938	0.86	0.52
2.43	11	2	30	28	0.282	0.938	0.85	0.51
2.54	11	1	31	28	0.282	0.969	0.92	0.52
2.56	11	0	32	28	0.282	1.000	1.00	0.53
2.58	10	0	32	29	0.256	1.000	1.00	0.52
2.65	9	0	32	30	0.231	1.000	1.00	0.51
2.89	8	0	32	31	0.205	1.000	1.00	0.51
3.03	7	0	32	32	0.179	1.000	1.00	0.50
3.10	6	0	32	33	0.154	1.000	1.00	0.49
3.15	5	0	32	34	0.128	1.000	1.00	0.48
3.26	4	0	32	35	0.103	1.000	1.00	0.48
3.43	3	0	32	36	0.077	1.000	1.00	0.47
3.61	2	0	32	37	0.051	1.000	1.00	0.46
3.75	1	0	32	38	0.026	1.000	1.00	0.45

4.39	0	0	32	39	0.000	1.000	-	0.45

Supplementary Table 6

Data analysis from Receiver operating characteristic curve (ROC) to obtain the optimal diagnostic cut-off value of $\delta_{DOB}{}^{18}O$ (‰) in breath CO_2 between pre-diabetes (PD) and type 2 diabetes (T2D). The selected optimal cut-off value has been highlighted. TP : True positive rate, FP : False positive rate, TN : True negative rate, FN : False negative rate.

T2D vs PD	TP	FP	TN	FN	Sensitivity	Specificity	Predictive Value (+)	Predictive Value (-)
-1.55	48	38	1	0	1.000	0.026	0.56	1.00
-1.19	48	37	2	0	1.000	0.051	0.56	1.00
-0.86	48	36	3	0	1.000	0.077	0.57	1.00
-0.64	48	35	4	0	1.000	0.103	0.58	1.00
0.88	48	34	5	0	1.000	0.128	0.59	1.00
1.07	48	33	6	0	1.000	0.154	0.59	1.00
1.11	48	32	7	0	1.000	0.179	0.60	1.00
1.18	48	31	8	0	1.000	0.205	0.61	1.00
1.42	48	30	9	0	1.000	0.231	0.62	1.00
1.44	48	29	10	0	1.000	0.256	0.62	1.00
1.46	48	28	11	0	1.000	0.282	0.63	1.00
1.55	48	27	12	0	1.000	0.308	0.64	1.00
1.57	48	26	13	0	1.000	0.333	0.65	1.00
1.67	48	25	14	0	1.000	0.359	0.66	1.00
1.72	48	24	15	0	1.000	0.385	0.67	1.00
1.73	48	23	16	0	1.000	0.410	0.68	1.00
1.74	48	22	17	0	1.000	0.436	0.69	1.00
1.82	47	22	17	1	0.979	0.436	0.68	0.94
1.86	47	21	18	1	0.979	0.462	0.69	0.95
1.95	47	20	19	1	0.979	0.487	0.70	0.95

1.98	47	19	20	1	0.979	0.513	0.71	0.95
2.01	47	18	21	1	0.979	0.538	0.72	0.95
2.04	47	17	22	1	0.979	0.564	0.73	0.96
2.08	46	17	22	2	0.958	0.564	0.73	0.92
2.33	46	16	23	2	0.958	0.590	0.74	0.92
2.44	46	15	24	2	0.958	0.615	0.75	0.92
2.44	46	14	25	2	0.958	0.641	0.77	0.93
2.48	46	13	26	2	0.958	0.667	0.78	0.93
2.55	46	12	27	2	0.958	0.692	0.79	0.93
2.55	46	11	28	2	0.958	0.718	0.81	0.93
2.57	46	10	29	2	0.958	0.744	0.82	0.94
2.59	46	9	30	2	0.958	0.769	0.84	0.94
2.62	46	8	31	2	0.958	0.795	0.85	0.94
2.66	46	7	32	2	0.958	0.821	0.87	0.94
2.67	46	6	33	2	0.958	0.846	0.88	0.94
2.74	46	5	34	2	0.958	0.872	0.90	0.94
2.76	46	4	35	2	0.958	0.897	0.92	0.95
2.77	46	3	36	2	0.958	0.923	0.94	0.95
2.80	45	3	36	3	0.938	0.923	0.94	0.92
2.87	44	3	36	4	0.917	0.923	0.94	0.90
2.90	43	3	36	5	0.896	0.923	0.93	0.88
3.19	42	3	36	6	0.875	0.923	0.93	0.86
3.22	41	3	36	7	0.854	0.923	0.93	0.84
3.44	40	3	36	8	0.833	0.923	0.93	0.82
3.54	39	3	36	9	0.813	0.923	0.93	0.80
3.55	38	3	36	10	0.792	0.923	0.93	0.78
3.69	37	3	36	11	0.771	0.923	0.93	0.77
3.96	37	2	37	11	0.771	0.949	0.95	0.77
4.11	36	2	37	12	0.750	0.949	0.95	0.76
4.14	35	2	37	13	0.729	0.949	0.95	0.74
4.23	34	2	37	14	0.708	0.949	0.94	0.73
4.42	33	2	37	15	0.688	0.949	0.94	0.71

4.53	32	2	37	16	0.667	0.949	0.94	0.70
4.73	31	2	37	17	0.646	0.949	0.94	0.69
4.88	31	1	38	17	0.646	0.974	0.97	0.69
5.02	30	1	38	18	0.625	0.974	0.97	0.68
5.18	29	1	38	19	0.604	0.974	0.97	0.67
5.23	28	1	38	20	0.583	0.974	0.97	0.66
5.27	28	0	39	20	0.583	1.000	1.00	0.66
5.42	27	0	39	21	0.563	1.000	1.00	0.65
5.47	26	0	39	22	0.542	1.000	1.00	0.64
5.61	25	0	39	23	0.521	1.000	1.00	0.63
5.84	24	0	39	24	0.500	1.000	1.00	0.62
6.01	23	0	39	25	0.479	1.000	1.00	0.61
6.11	22	0	39	26	0.458	1.000	1.00	0.60
6.11	21	0	39	27	0.438	1.000	1.00	0.59
6.18	20	0	39	28	0.417	1.000	1.00	0.58
6.21	19	0	39	29	0.396	1.000	1.00	0.57
6.25	18	0	39	30	0.375	1.000	1.00	0.57
6.47	17	0	39	31	0.354	1.000	1.00	0.56
6.49	16	0	39	32	0.333	1.000	1.00	0.55
6.54	15	0	39	33	0.313	1.000	1.00	0.54
6.59	14	0	39	34	0.292	1.000	1.00	0.53
6.74	13	0	39	35	0.271	1.000	1.00	0.53
6.81	12	0	39	36	0.250	1.000	1.00	0.52
6.88	11	0	39	37	0.229	1.000	1.00	0.51
7.02	10	0	39	38	0.208	1.000	1.00	0.51
7.04	9	0	39	39	0.188	1.000	1.00	0.50
7.20	8	0	39	40	0.167	1.000	1.00	0.49
7.42	7	0	39	41	0.146	1.000	1.00	0.49
7.55	6	0	39	42	0.125	1.000	1.00	0.48
7.80	5	0	39	43	0.104	1.000	1.00	0.48
7.87	4	0	39	44	0.083	1.000	1.00	0.47
8.01	3	0	39	45	0.063	1.000	1.00	0.46

8.11	2	0	39	46	0.042	1.000	1.00	0.46
8.64	1	0	39	47	0.021	1.000	1.00	0.45
8.87	0	0	39	48	0.000	1.000	-	0.45

Supplementary Table 7

Data analysis from Receiver operating characteristic curve (ROC) to obtain the optimal diagnostic cut-off value of $\delta_{DOB}{}^{18}O$ (‰) in breath CO_2 between pre-diabetes (PD) and non-diabetic control (NDC). The selected optimal cut-off value has been highlighted. TP : True positive rate, FP : False positive rate, TN : True negative rate, FN : False negative rate.

PD vs NDC	TP	FP	TN	FN	Sensitivity	Specificity	Predictive Value (+)	Predictive Value (-)
-6.41	39	31	1	0	1.000	0.031	0.56	1.00
-5.79	39	30	2	0	1.000	0.063	0.57	1.00
-5.42	39	29	3	0	1.000	0.094	0.58	1.00
-4.93	39	28	4	0	1.000	0.125	0.58	1.00
-4.83	39	27	5	0	1.000	0.156	0.59	1.00
-4.72	39	26	6	0	1.000	0.188	0.60	1.00
-4.66	39	25	7	0	1.000	0.219	0.61	1.00
-4.64	39	24	8	0	1.000	0.250	0.62	1.00
-4.60	39	23	9	0	1.000	0.281	0.63	1.00
-3.93	39	22	10	0	1.000	0.313	0.64	1.00
-3.47	39	21	11	0	1.000	0.344	0.65	1.00
-3.44	39	20	12	0	1.000	0.375	0.66	1.00
-3.31	39	19	13	0	1.000	0.406	0.67	1.00
-3.14	39	18	14	0	1.000	0.438	0.69	1.00
-2.81	39	17	15	0	1.000	0.469	0.70	1.00
-2.76	39	16	16	0	1.000	0.500	0.71	1.00
-2.65	39	15	17	0	1.000	0.531	0.72	1.00
-2.61	39	14	18	0	1.000	0.563	0.74	1.00

-2.47	39	13	19	0	1.000	0.594	0.75	1.00
-2.47	39	12	20	0	1.000	0.625	0.77	1.00
-2.45	39	11	21	0	1.000	0.656	0.78	1.00
-2.22	39	10	22	0	1.000	0.688	0.80	1.00
-2.03	39	9	23	0	1.000	0.719	0.81	1.00
-1.87	39	8	24	0	1.000	0.750	0.83	1.00
-1.73	39	7	25	0	1.000	0.781	0.85	1.00
-1.57	39	6	26	0	1.000	0.813	0.87	1.00
-1.55	38	6	26	1	0.974	0.813	0.86	0.96
-1.54	38	5	27	1	0.974	0.844	0.88	0.96
-1.47	38	4	28	1	0.974	0.875	0.91	0.97
-1.19	37	4	28	2	0.949	0.875	0.90	0.93
-1.14	37	3	29	2	0.949	0.906	0.93	0.93
-0.86	36	3	29	3	0.923	0.906	0.92	0.91
-0.64	35	3	29	4	0.897	0.906	0.92	0.88
-0.44	35	2	30	4	0.897	0.938	0.95	0.88
0.58	35	1	31	4	0.897	0.969	0.97	0.88
0.88	34	1	31	5	0.872	0.969	0.97	0.86
1.07	33	1	31	6	0.846	0.969	0.97	0.84
1.11	32	1	31	7	0.821	0.969	0.97	0.81
1.18	31	1	31	8	0.795	0.969	0.97	0.79
1.42	30	1	31	9	0.769	0.969	0.97	0.77
1.44	29	1	31	10	0.744	0.969	0.97	0.75
1.46	28	1	31	11	0.718	0.969	0.97	0.74
1.55	27	1	31	12	0.692	0.969	0.96	0.72
1.57	26	1	31	13	0.667	0.969	0.96	0.70
1.67	25	1	31	14	0.641	0.969	0.96	0.69
1.72	24	1	31	15	0.615	0.969	0.96	0.67
1.73	23	1	31	16	0.590	0.969	0.96	0.66
1.74	22	1	31	17	0.564	0.969	0.96	0.64
1.75	22	0	32	17	0.564	1.000	1.00	0.65
1.86	21	0	32	18	0.538	1.000	1.00	0.64

1.95	20	0	32	19	0.513	1.000	1.00	0.63
1.98	19	0	32	20	0.487	1.000	1.00	0.61
2.01	18	0	32	21	0.462	1.000	1.00	0.60
2.04	17	0	32	22	0.436	1.000	1.00	0.59
2.33	16	0	32	23	0.410	1.000	1.00	0.58
2.44	15	0	32	24	0.385	1.000	1.00	0.57
2.44	14	0	32	25	0.359	1.000	1.00	0.56
2.48	13	0	32	26	0.333	1.000	1.00	0.55
2.55	12	0	32	27	0.308	1.000	1.00	0.54
2.55	11	0	32	28	0.282	1.000	1.00	0.53
2.57	10	0	32	29	0.256	1.000	1.00	0.52
2.59	9	0	32	30	0.231	1.000	1.00	0.51
2.62	8	0	32	31	0.205	1.000	1.00	0.51
2.66	7	0	32	32	0.179	1.000	1.00	0.50
2.67	6	0	32	33	0.154	1.000	1.00	0.49
2.74	5	0	32	34	0.128	1.000	1.00	0.48
2.76	4	0	32	35	0.103	1.000	1.00	0.48
2.77	3	0	32	36	0.077	1.000	1.00	0.47
3.96	2	0	32	37	0.051	1.000	1.00	0.46
4.88	1	0	32	38	0.026	1.000	1.00	0.45
5.27	0	0	32	39	0.000	1.000	-	0.45

Supplementary Table 8

Important diagnostic parameters corresponding to cut-off values of ΔCA activity (U/min/mL) and $\delta_{DOB}{}^{18}O$ (‰) by ICOS method for screening non-diabetic control (NDC), pre-diabetes (PD) and type 2 diabetes (T2D) individuals. AUC: area under the curve; PPV: positive predictive value; NPV: negative predictive value.

**Groups**	**Cut-off points of ΔCA activity (U/min/mL)**	**Sensitivity**	**Specificity**	**PPV**	**NPV**	**AUC**	**Accuracy**
NDC vs PD	-1.31	100%	90.6%	93.0%	100%	0.945	95.77%
PD vs T2D	3.15	91.7%	94.9%	96.0%	90.0%	0.957	93.10%

**Groups**	**Cut-off points of $\delta^{18}O$ (‰)**	**Sensitivity**	**Specificity**	**PPV**	**NPV**	**AUC**	**Accuracy**
NDC vs PD	-1.14	94.9%	90.6%	93.0%	93.0%	0.977	92.9%
PD vs T2D	2.77	95.8%	92.3%	94.0%	95.0%	0.957	94.2%
